# Knowledge, attitudes, and perceptions of the multi-ethnic population of the United Arab Emirates on genomic medicine and genetic testing

**DOI:** 10.1186/s40246-023-00509-0

**Published:** 2023-07-15

**Authors:** Azhar T. Rahma, Bassam R. Ali, George P. Patrinos, Luai A. Ahmed, Iffat Elbarazi, Aminu S. Abdullahi, Mahanna Elsheik, Maram Abbas, Farah Afandi, Aisha Alnaqbi, Fatma Al Maskari

**Affiliations:** 1 Institute of Public Health, College of Medicine and Health Sciences, United Arab Emirates University, Al-Ain, UAE; 2 Department of Genetics and Genomics, College of Medicine and Health Sciences, United Arab Emirates University, Al-Ain, UAE; 3 Department of Pharmacy, School of Health Sciences, University of Patras, Patras, Greece; 4 Zayed Center for Health Sciences, United Arab Emirates University, Al-Ain, Abu Dhabi, UAE; 5 Bloomberg School of Public Health, Johns Hopkins University, Baltimore, MD USA; 6 Department of Clinical Pharmacy and Therapeutics, Dubai Pharmacy College for Girls, Dubai, UAE; 7 USF Biotechnology, Morsani College of Medicine, University of South Florida, Tampa, FL USA; 8 Department of Biochemistry, College of Medicine and Health Sciences, United Arab Emirates University, Al-Ain, UAE

**Keywords:** Genomic literacy, Genetic tests, Personalized medicine, Disclosure, Implementation, United Arab Emirates

## Abstract

**Introduction:**

The adoption and implementation of genomic medicine and pharmacogenomics (PGx) in healthcare systems have been very slow and limited worldwide. Major barriers to knowledge translation into clinical practice lie in the level of literacy of the public of genetics and genomics. The aim of this study was to assess the knowledge, attitudes, and perceptions of the United Arab Emirates (UAE) multi-ethnic communities toward genomic medicine and genetic testing.

**Method:**

A cross-sectional study using validated questionnaires was distributed to the participants. Descriptive statistics were performed, and multivariable logistic regression models were used to identify factors associated with knowledge of genomics.

**Results:**

757 individuals completed the survey. Only 7% of the participants had a good knowledge level in genetics and genomics (95% CI 5.3–9.0%). However, 76.9% of the participants were willing to take a genetic test if their relatives had a genetic disease. In addition, the majority indicated that they would disclose their genetic test results to their spouses (61.5%) and siblings (53.4%).

**Conclusions:**

This study sets the stage for the stakeholders to plan health promotion and educational campaigns to improve the genomic literacy of the community of the UAE as part of their efforts for implementing precision and personalized medicine in the country.

**Supplementary Information:**

The online version contains supplementary material available at 10.1186/s40246-023-00509-0.

## Introduction

The recent and rapid advances in genomic sciences and medicine have pushed the fields of pharmacogenomics (PGx) and genomic medicine into the limelight with major efforts at implementing precision medicine. PGx combines the fields of pharmacology and genomics and deals with the study of how an individual's genetic composition affects their response to different drugs and medications [1]. The genetic make of the individual plays a significant role in drug uptake, interactions, breakdown, and clearance, processes that underpin drug response [1, 2].

Since the genomic profile of each individual is unique, the ultimate goal of genomic medicine and PGx is to allow the individualization of diagnosis, analysis, and treatment of various diseases based on patient genetic makeup [3]. Commercialization of this application is known as personalized and precision medicine and is thought to have the potential to optimize drug therapy and improve outcomes, while reducing side effects and costs [3–5]. This is thought to hold great potential, particularly with potentially toxic medications [6–8].

The field of pharmacogenomics is growing at an unprecedented pace, with more than 350 drugs incorporated in the US Food and Drug Administration’s (FDA) list of reviewed medications [6–8]. However, the implementation in healthcare systems globally has been very sluggish, variable and limited. Different studies suggested that one of the major barriers for knowledge translation into clinical practice lies in the level of literacy of the general public [9]. In particular, the distinctive nature of genomic testing raised specific social, ethical, religious, and cultural implications [10–13] .

In the Middle East and North Africa region, little is known about the level of genetic and genomic literacy of the public. However, recently, several Middle Eastern countries have begun to implement genomic research initiatives. For example, recent studies from Saudi Arabia emphasized the importance of knowledge in the field [14]. In the UAE, the field of pharmacogenomics and genomics is evolving at a good pace ever since government support was declared. A landmark study examines the potential benefits of implementing pharmacogenomic (PGx) testing in cardiovascular disease treatment in the UAE revealed a significant proportion of participants with genotypes suggesting suboptimal drug metabolism.

{Al-Mahayri, 2022 #616}. Several important initiatives were launched including the Emirati Genome Project, which intending on using genomic data to provide more personalized healthcare for citizens of the UAE [15]. Several studies have been conducted to evaluate knowledge and attitudes of different groups toward PGx and genomic medicine in the UAE that revealed positive attitudes and a high degree of interest from various stakeholders, but with insufficient knowledge and training [12, 13, 16, 17]. However, no research has been carried out to evaluate this at the general public level. This is critical, because in order to exploit the potential of genomic medicine, it is necessary to identify the levels of health and genomic literacy of all community sectors including the public [18, 19]. The UAE population is distinctly diverse and heterogeneous with about 90% of the UAE residents are expatriates.

The aim of this study was to assess the knowledge, attitudes, and perceptions of the UAE community toward genomic medicine and genetic testing in an attempt to identify, and potentially provide recommendations to close the gaps in the genomic awareness and knowledge of the recipients of healthcare provisions in the country. Furthermore, it attempts to identify factors related to a favorable attitude toward the implementation of genomic medicine in the UAE, with its highly heterogenous and multi-ethnic population.

## Method

### Study design, tool, and sampling

A cross-sectional study was conducted based on validated questionnaires on the knowledge, attitudes, and perceptions of genetic testing, PGx, and personalized medicine [20–22]. The study population included residents and citizens of the UAE. The survey was established and reviewed for structure, content, and readability by subject experts ensuring it was easy to understand with accurate information. The survey was piloted among a sample of 57 participants and adjusted based on the feedback received from the experts and pilot sample.

The survey was distributed online and in person in both Arabic and English languages, and we followed the Checklist for Reporting Results of Internet E-Surveys (CHERRIES)[23]. Participants were recruited using a combination of methods including email, WhatsApp, LinkedIn, Facebook, and other social media platforms. This process includes contacting potential participants by email, exchanging information about research studies and inviting them to participate. Additionally, a post was published on social media platforms announcing the research study and providing details on how interested individuals could participate. Moreover, we asked participants to invite their contacts to participate in the study (snowballing). Participants were selected based on inclusion criteria of the study. Individuals who met established criteria and expressed an interest in participating were selected to participate in the study. Snowball sampling was utilized to ensure a diverse and representative sample to enhance the generalizability of the research findings. The calculated sample size (based on the cross-sectional survey formula and WHO calculator) was 383. From the literature, we estimated an average response rate of 69%; therefore, our final sample size was 502. This study was approved by the Social Science Research Ethics Committee of United Arab Emirates University (UAEU) ERS_2017_5671. Survey participants were provided with an information sheet and a consent form stating a guarantee of anonymity and their right to withdraw from the study at any time. The survey was administered from April to May 2022.

The questionnaire was sectioned into 1) questions on demographic data including age, gender, and socioeconomic status, 2) general knowledge questions on genetic testing and genomic medicine in the UAE, 3) attitudes, perceptions, and views toward genetic testing and personalized medicine. The score of Flesch Reading Ease test for the survey was 60; moreover, the Flesch-Kincaid grade level test was 70.

### Data analysis

Descriptive statistics were performed to describe the study participants based on demographic characteristics, their responses to specific questions, their knowledge and preferences, attitudes toward genomics, as well as their sources of information. Median and interquartile range were used to summarize age, a continuous variable, owing to the nature of its distribution being skewed as evaluated using the Shapiro–Wilk test. All other variables, being categorical, were summarized using frequencies and percentages.

A variable for knowledge was generated as a dichotomous variable – good knowledge and poor knowledge. This variable was computed by first assigning a score to each of the five questions on knowledge. For any knowledge question answered correctly, a score of one was assigned, if otherwise a score of zero was assigned. These scores were summed up for all the five questions for each participant. The individual sums were then converted into percentages. These percentage scores were further categorized into – good knowledge (a percentage score of 70 and above) and poor knowledge (a percentage score of less than 70) as reported in other studies [13, 24].

Furthermore, univariate and multivariable logistic regression models were used to identify factors associated with knowledge of genomics among the participants. SPSS version 28 was used for the analysis (IBM Corp. Released 2021. IBM SPSS Statistics for Windows, version 28.0. Armonk, NY: IBM Corp).

## Results

### Demographic characteristics of the participants

757 completed the survey, the demographic characteristics of the participants are summarized in Table 1. On average, the participants were aged 34 years (interquartile range = 17). The participants were mostly females (77.1%), resident of Abu Dhabi Emirate (66.1%), and employed (60.2%). More than half (57.6%) were non-Emiratis and 54.7% were married. The majority of the respondents (86%) had at least a bachelor’s degree. Detailed description of nationalities and ethnicities is included in the supplementary document.

**Table 1:** Demographic characteristics of the participants (N = 757)

Characteristics	Frequency	Percentage
Age in years, median (IQR)	34	(IQR = 17)
*Gender*		
Female	584	77.1
Male	173	22.9
*Nationality*		
Emirati	321	42.4
Non-Emirati	436	57.6
*Marital status*		
Single	320	42.3
Married	414	54.7
Divorced/separated/widowed	23	3.0
*Place of residence*		
Abu-Dhabi	476	66.1
Dubai	107	14.9
Sharjah	50	6.9
Others	87	12.1
*Highest education*		
High school	82	10.8
Diploma	39	5.2
Bachelors	393	52
Masters	173	22.9
PhD	50	6.6
Others	19	2.5
*Employment status*		
Unemployed	301	39.8
Employed	456	60.2

Some variables may not add to 757 due to missing data

### Knowledge of genomics and its associated factors

**Fig. 1:**
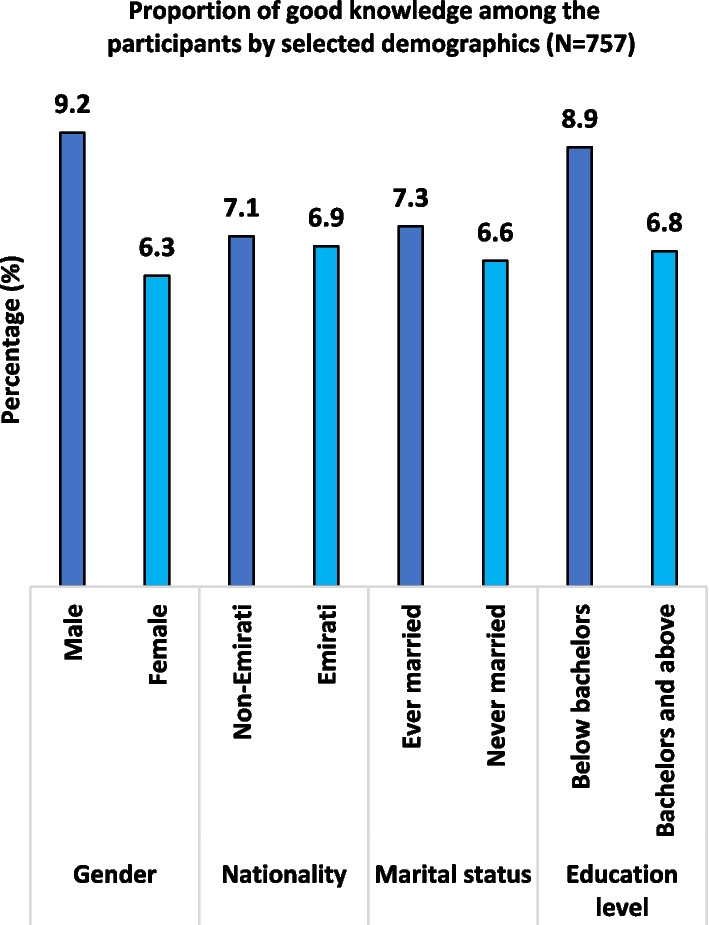
Proportion of good knowledge among the participants by selected demographics

The overall proportion of good knowledge in genetics and genomics among the participants was 7% (95% CI 5.3–9.0%). Moreover, some differences in the proportion of the participants with good knowledge were observed across some characteristics (Fig. 1). Male had more knowledge than their female counterparts (9.2% versus 6.3%). Participants with at least a bachelor's degree exhibited more knowledge than those with lower education levels (8.9% versus 6.8%). Slight differences were also seen between non-Emiratis (7.1%) and Emiratis (6.9%) as well as between ever-married (7.3%) and never-married (6.6%) participants. However, none of these observed differences were not statistically significant.

Furthermore, having an existing chronic condition was seen to be statistically significantly associated with having good knowledge as a multivariable logistic regression model reveals that participants who had a chronic condition were at least two times more likely (adjusted odds ratio = 2.25, p = 0.043) to have good knowledge than those that did not have a chronic disease (Table 2).

**Table 2:** Factors significantly associated with good knowledge of genomics

Predictor	Crude OR (95%CI)	P-value	Adjusted (95% CI)	**P-value**
*Having chronic condition*				
No	1		1	
Yes	3.38 (1.82–6.29)*	< 0.001	2.25 (1.03–4.92)*	0.043
*Taking long-term medication*				
No	1		1	
Yes	2.77 (1.55–4.95)*	< 0.001	2.02 (0.93–4.40)	0.076
*Willingness to buy online genetic test kit*				
No	1		1	
Yes	0.65 (0.29–1.45)	0.292	0.70 (0.31–1.58)	0.386
Not sure	2.09 (1.09–4.04)*	0.027	1.97 (0.95–4.01)	0.069

This multivariable model includes only significant predictors from the univariate analysis. Other variables explored but found to be non-significant include age, gender, marital status, education level, and nationality

*Indicates statistical significance; OR—odds ratio; CI—confidence interval

Based on participants’ knowledge of genomics (Additional file 1), the majority (81.8%) knew about what is meant by DNA or genetic materials; about 98% knew about the various samples used for genetic testing, even though none of the participants mentioned all the available sample materials on the list; 95.6% knew at least one of the various places where genetic tests could be performed, although only a minority (43.7%) were able to mention all the available places; and 90.1% got the correct percentage of ladies with breast cancer based on a pictogram designed to test their knowledge. However, the participants reported very poor knowledge (15.5%) of university programs in the UAE that offer genomic medicine training.

Regarding the open-ended question about "what comes to the participants’ mind when they hear genomic medicine,” we alluded the following salient codes from 744 responses: prevention, diagnosis, and treatment of diseases in general and hereditary disease (104 mentions) in specific were top-mentioned answer (166 mentions), followed by mentioning genes (113 mentions) and DNA (112 mentions) in the context of using DNA and genes for medicine as well as genetic engineering. Other words that had been used by our cohort are future (20 mentions), new (13 mentions), medications (9 mentions), cancer (7 mentions), technology (6 mentions), marriage, children, and parents (all 5 mentions). On contrast 36 mentions were for nothing, or never heard of it or do not understand the term, however they were stating that they are interested in knowing more about genomic medicine. Some unexpected answers included: my friend, nuclear medicine, Pfizer and feeling anxious (Additional file 2).

#### Attitudes toward genetic tests and genetic research

About three-quarters (76.9%) of the participants were willing to take a genetic test if their relatives had genetic diseases (Additional file 3). While a few of the participants believed genetic tests were painful (3.4%), the majority of them thought genetic tests were costly (64.9%) and took a long time for the results to be released (59.5%). With regard to participation in genetic research, the majority (59.1%) would like to participate in genetic research and were willing to get genetic test results if participated in genetic research (79.1%). The majority said they would disclose their genetic test results to their spouses (61.5%) and siblings (53.4%), whereas only a few hinted they would disclose their results to their parents (13.5%).

A negligible proportion of the participants previously participated in genetic research (1.8%). Moreover, only about one-third (32%) of the Emirati participants reported donating sample to the Emirati Genome Project. When asked about their reasons for participating in genetic research, the following codes were pinpointed as visualized in word cloud (Additional file 4) derived to participate because of being sick with genetic disease or having a family member diagnosed with chronic disease (25 mentions), others wanted to know their disease risk score to diseases and how their body will response to medications (24 mentions). Other codes, including the desire to help and benefit the UAE community and humanity in general and doing good deeds as instructed by religion, moreover, help protecting their children (19 mentions). In addition, they want to benefit the research arena and help shaping the future (14 mentions). We used inductive qualitative analysis to examine reasons for not participating in genetic research. Codes included lack of knowledge, privacy concerns, time constraints, fear of results, and fear of being cloned.

### Effects of religion, culture, and genetic testing costs upon their attitudes

**Fig. 2:**
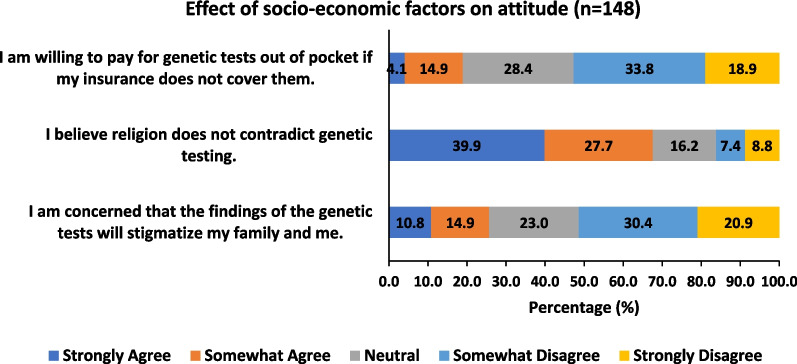
Effect of socioeconomic factors on attitude

Looking at the effect of religion on our cohort ‘s attitude, we found that more than half of our cohort (67.6%) agreed that religion does not contradict genetic testing; however, 8.8% and 7.4% strongly disagree and somewhat disagree with this statement, respectively (Fig. 2). Nevertheless, exploring the effect of culture only 25.7% were concerned with the stigmatization of themselves or their families based on the results of the genetic tests. Moreover, only 19.3% of our cohort were willing to pay out of pocket for the genetic tests. (Fig. 2).

### Attitudes toward consanguinity

Slightly more than one quarter (27.8%) of the participants opined that a person with a genetic disease or at risk of one could marry his/her cousin. This opinion was observed to be more frequent among the males (35.3%) than the females (24.7%), the non-Emiratis (29.7%) than the Emiratis (24.4%), and among the married (29.9%) than the unmarried (25%). Moreover, participants with the same attitude tend to have better knowledge of genomics than those who were not in favor (15.6% among those with good knowledge versus 11.4% among those with poor knowledge).

#### Genetic tests and counseling preferences

About four in every five participants (80.6%) preferred government facilities in the UAE for their genetic tests compared to private facilities (34.1%) and sending the sample abroad (26%) (Additional file 5). A slight majority (54.7%) preferred a physician for their genetic counseling followed by a genetic counselor (41.9%). Majority preferred face-to-face counseling session (87.8%) to virtual session (12.2%). Videos (51.4%) were reported to be the preferred educational method for a genetic counseling session. More than half of the participants (57.3%) preferred blood as the sample material to be collected for a genetic test.

#### Sources of information

**Fig. 3:**
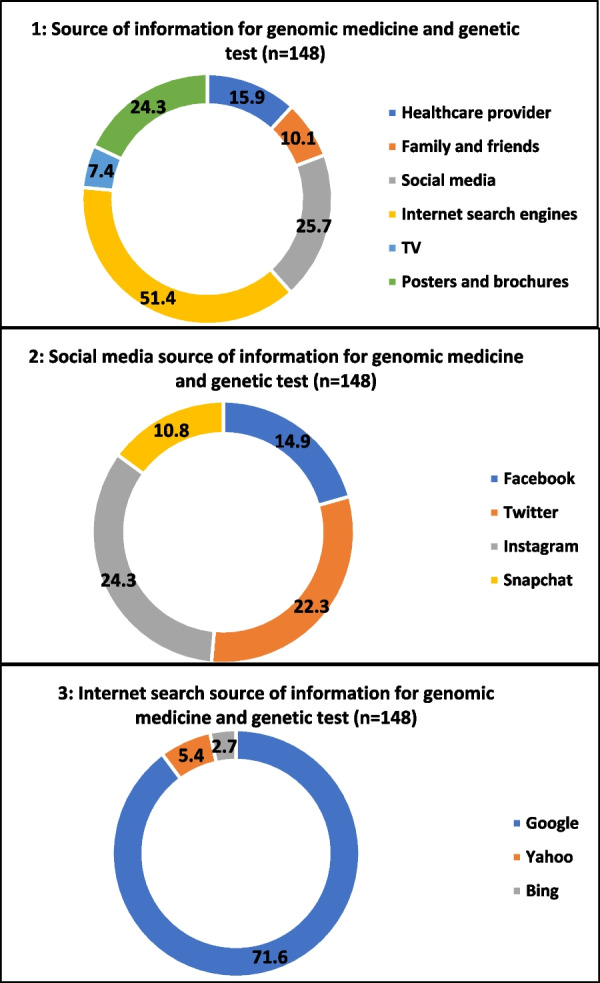
Participants’ sources of information

Overall, internet search engines turned out to be the most common source of information for genomic medicine and genetic test among the participants with more than half of them (51.4%) reporting it (Fig. 3). This was followed by social media (25.7%) and posters and brochures (24.3%). Among the internet search engine sources, Google was the most common (71.6%) as Yahoo (5.4%) and Bing (2.7%) were rarely reported to be used by the participants while Instagram (24.3%) was the most common among the social media sources, followed by Twitter (22.3%), Facebook (14.9%) and Snapchat (10.8%).

## Discussion

This cohort showed insufficient knowledge of genomic medicines and their elements among the UAE population, as only 7% of the participants had a good knowledge level in genetics and genomics Culture and religion had little impact on the participants' attitudes, as more than half of our cohort (67.6%) agreed that religion does not contradict genetic testing. Moreover, only 25.7% were concerned with the stigmatization of themselves or their families based on the results of the genetic tests.

As part of the MENA region, UAE still suffers from the suboptimal practice of newly merged personalized medicine [25]. One of the obstacles in implementing novel practices such as genomic medicine is public and healthcare professional awareness [19]. Unfortunately, a minority of participants in this study were aware of the basics of genomic medicine and its services in UAE, with a favored present to male over female participants; this result was inferior to (Basima et al. 2020) study in Jordan, which revealed that about 43% of the study participants were aware of genetics with the favor to females [24]. Nevertheless, other studies worldwide, such as (Susanne B. Haga et al. 2013) in the USA, showed that most respondents had some knowledge [26]. However, because various groups were examined, different questionnaires and calculation techniques were employed in earlier studies, comparing the percentage in the current study with that in those earlier investigations is challenging.

Remarkably, participants with chronic disease had good knowledge scores; this could be explained by the fact that sick people are straining to seek more medical information than healthy ones. The opposite was found in other studies, which showed that participants with poor or fair health have lower levels of awareness about genetic testing than those with good health[27, 28].

Studies revealed that better knowledge of genetics correlates with higher education levels [31]. In this study, respondents with bachelor's degrees or higher education certificates obtained better knowledge scores aligned with other studies in the same field [24, 32]. Enhancing healthcare providers knowledge in genetic testing and personalized medicine is expected to improve the clinical application of precision medicine with better patient comprehension regarding the impact of genomics on pharmacotherapy, decreased healthcare expenses, enhanced quality of life, and improved communication between patients and healthcare providers. {Jarrar, 2022 #540}{Rahma, 2021 #474}.

Regarding the attitude, most participants were willing to perform genetic testing if any family member was diagnosed with a genetic disease, which was in line with the growing body of studies worldwide supporting a positive attitude among populations toward genetic testing [29, 30]. Despite the positive attitude toward genetic testing, only a minority of our participants took part in the Emirati genome project; a similar attitude was noticed in the (Qun Wang1 et al.) study in China, where 45% of the participant detained positive views toward genetic testing, but only 4% have used it [28].

Middle Eastern cultures have severely stigmatized perceptions of health issues[31]. A quarter of this study's participants were concerned about the social stigma of themselves or their families due to the results of the genetic tests, which is almost the same scenario in (Almomani BA. et al.) study [24]. About 16% of participants did not agree with the statement “no disagreement between religion and genetic testing,” which is a concerning percentage if we consider how pivotal the religious and spiritual views on accepting genetic testing.

Noticeably, most participants preached that a person with a genetic disease or at risk of one should not marry his/her cousin. Considering that Arab nations are among the world's highest proportions of consanguineous marriages [32] and the impact of the rate of consanguinity on the beliefs of genetic diseases and consanguinity [33].

The physician was the preferred person to advise regarding genetic tests. Based on our previous work to develop a roadmap for the implantation of genomic medicine in UAE, stakeholders articulated that there is shortage of genetic counsellors and contemplated that as one of the barriers for the implantation of genomic medicine at UAE. {Rahma, 2021 #329}However, many studies showed that patients' preferences regarding which medical specialist should do these tests vary, but most prefer receiving these services from a trusted healthcare provider [34]. Most participants preferred face-to-face counseling sessions, which aligns with ( Nina Beri et al.) study on the preferences for in-person disclosure [35]. Interestingly, governmental medical facilities took the first place as the preferred place to perform genetic testing, reflecting participants' trust in these facilities.

Overall, internet search engines turned out to be the most common source of information for genomic medicine and genetic test among the participants, which is aligned with (Maria et al.) study about the growing role of the internet and media in health-related information [28]. In comparison, a fourth of participants utilized social media to get genetic information, which is supported by (D. Mansour et al.) study about the role of social media in the Gulf Cooperation Council (GCC), which includes the following countries: the United Arab Emirates, Bahrain, Kuwait, Oman, Qatar, and Saudi Arabia.[29].

This study's strengths are that it was the first study in the UAE that includes perceived knowledge of genomic medicine and PGx among community members. In addition to the inclusion of Emiratis and non-Emirati which expanded the outcomes to reflect all spheres of the community, which is helpful to the stakeholders occupied with the implementation of genomic medicine in the country. The limitation of our study originates from the inheritance bias of the cross-sectional study and the snowball sampling technique. Another weakness resides in the inadequate responses from the other six emirates of the UAE, which hinders the generalization of the study findings and results. The relatively small number of participants with a high level of knowledge in genomics may introduce a potential bias in the study; another limitation is that we did not ask about the type of religion, nor field of education in our cohort. Adopting a validated questionnaire with closed and open-ended questions in many languages will be of value for national and international comparison purposes and validation. Future studies can assess this knowledge gap by measuring the impact of health promotion campaigns related to genomic medicine on the genetic literacy of the population of the UAE.

## Conclusions

This study sets the stage for the stakeholders to plan health promotion and educational campaigns to improve genomic literacy of the community of UAE, which will speed the full implementation of the genomic medicine in the country. Moreover, we recommend that Department of Health and hospitals here to incorporate information about basics of genomic medicine and its services in their official social media accounts to be a trustful and validate source of information for the community. We also recommend that universities at UAE to launch programs for genetic counselors as physicians are overburden especially with the pandemics and genetic counselors will streamline the implementation process as well will aid the physicians. On the other hand, we urge the stakeholders to ensure the coverage of genetic tests under insurance companies. We recommend continuous assessment and exploration of the knowledge and the attitudes of the public and patients using a mixed method approach.

## Supplementary Information

**Additional file 1:** Summary of responses to knowledge-related questions

**Additional file 2:** Word cloud of answers to the open-ended question: “What comes to the participants’ mind when they hear genomic medicine”

**Additional file 3:** Attitudes toward genetic test and genetic research participation

**Additional file 4:** Why you will participate in genetic research or why you will not?

**Additional file 5:** Preferences for the genetic test and counseling

## Data Availability

The data used in this study are available upon reasonable request to the corresponding author.
