# Supplementary material for: Knowledge, attitudes, and perceptions of the multi-ethnic population of the United Arab Emirates on genomic medicine and genetic testing
Source: Hum Genomics. 2023 Jul 15;17:63. doi: 10.1186/s40246-023-00509-0 (PMC10349494; doi:10.1186/s40246-023-00509-0)
Supplement: Supplementary file 2 — Additional file 2: Word cloud of answers to the open-ended question: “What comes to the participants’ mind when they hear genomic medicine” [file 40246_2023_509_MOESM2_ESM.docx]

**
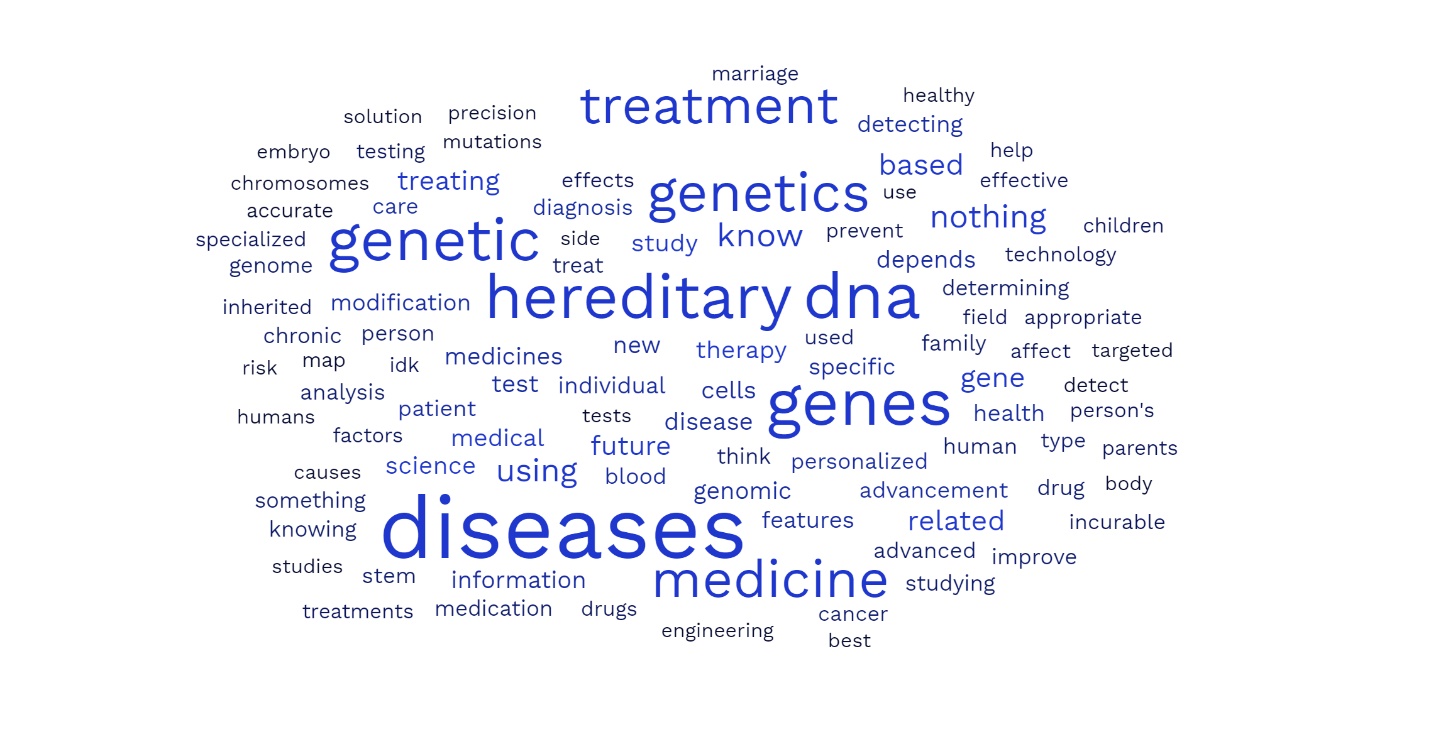
Additional file 2**: **Word cloud of answers to the open-ended question: “What comes to the participants’ mind when they hear genomic medicine”**
