# Supplementary material for: Knowledge, attitudes, and perceptions of the multi-ethnic population of the United Arab Emirates on genomic medicine and genetic testing
Source: Hum Genomics. 2023 Jul 15;17:63. doi: 10.1186/s40246-023-00509-0 (PMC10349494; doi:10.1186/s40246-023-00509-0)
Supplement: Supplementary file 3 — Additional file 3: Attitudes toward genetic test and genetic research participation [file 40246_2023_509_MOESM3_ESM.docx]

**Additional file 3: Attitudes toward genetic test and genetic research participation**
