# Supplementary figures and images for: Knowledge, attitudes, and perceptions of the multi-ethnic population of the United Arab Emirates on genomic medicine and genetic testing
Source: Hum Genomics. 2023 Jul 15;17:63. doi: 10.1186/s40246-023-00509-0 (PMC10349494; doi:10.1186/s40246-023-00509-0)

**Additional file 4: Why you will participate in genetic research or why you will not?**

**
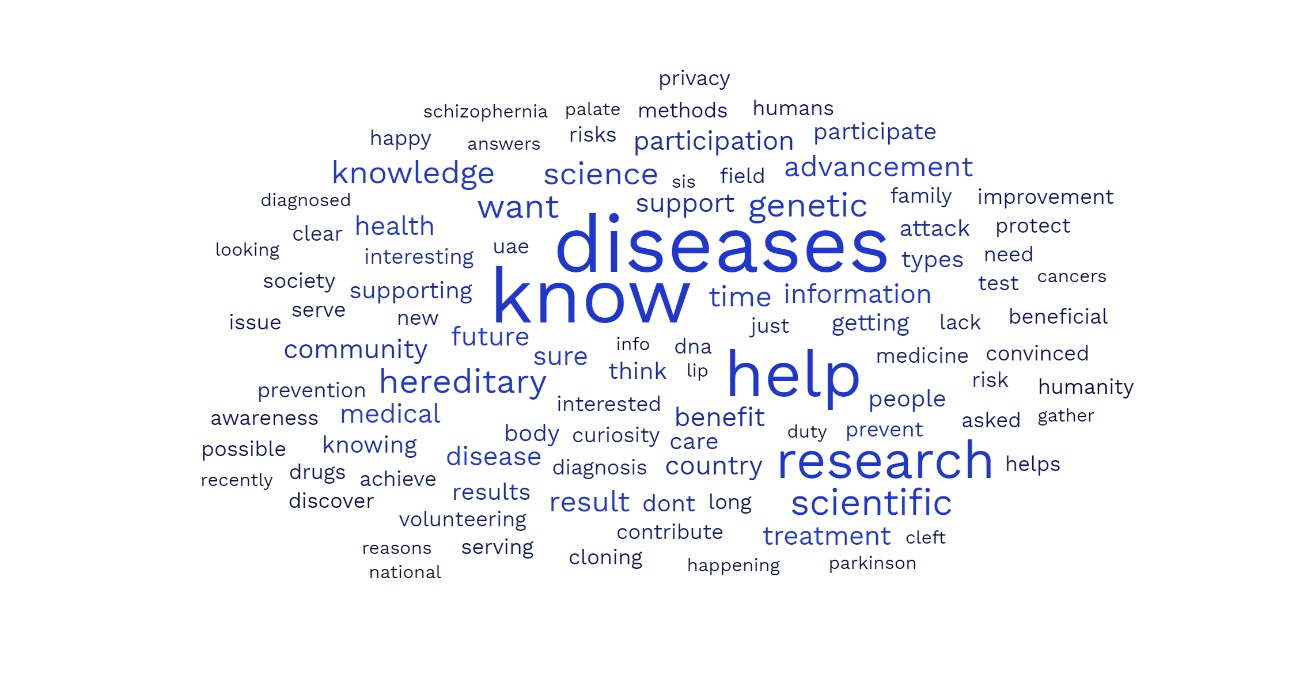
**

Supplement: Supplementary file 4 — Additional file 4: Why you will participate in genetic research or why you will not? [file 40246_2023_509_MOESM4_ESM.docx]
